# Application of the Chitooligosaccharides and Fluorescence Polarization Technique for the Assay of Active Lysozyme in Hen Egg White

**DOI:** 10.3390/biom14121589

**Published:** 2024-12-12

**Authors:** Liliya I. Mukhametova, Dmitry O. Zherdev, Sergei A. Eremin, Pavel A. Levashov, Hans-Christian Siebert, Yury E. Tsvetkov, Olga N. Yudina, Vadim B. Krylov, Nikolay E. Nifantiev

**Affiliations:** 1 Faculty of Chemistry, M.V. Lomonosov Moscow State University, Leninsky Gory 1/3, 119991 Moscow, Russia; liliya106@mail.ru (L.I.M.); me@zherderini.ru (D.O.Z.); eremin_sergei@hotmail.com (S.A.E.); levashov@yahoo.com (P.A.L.); 2 RI-B-NT—Research Institute of Bioinformatics and Nanotechnology, Schauenburger Str. 116, 24118 Kiel, Germany; hcsiebert@aol.com; 3 Laboratory of Glycoconjugate Chemistry, N.D. Zelinsky Institute of Organic Chemistry, Russian Academy of Sciences, Leninsky Prospect 47, 119991 Moscow, Russia; tsvetkov@ioc.ac.ru (Y.E.T.); olgay05@mail.ru (O.N.Y.); 4 Laboratory of Synthetic Glycovaccines, N.D. Zelinsky Institute of Organic Chemistry, Russian Academy of Sciences, Leninsky Prospect 47, 119991 Moscow, Russia

**Keywords:** lysozyme, activity, fluorescence polarization, fluorescence polarization assay

## Abstract

This study describes the applicability of the fluorescence polarization assay (FPA) based on the use of FITC-labeled oligosaccharide tracers of defined structure for the measurement of active lysozyme in hen egg white. Depending on the oligosaccharide chain length of the tracer, this method detects both the formation of the enzyme-to-tracer complex (because of lectin-like, i.e., carbohydrate-binding action of lysozyme) and tracer splitting (because of chitinase activity of lysozyme). Evaluation of the fluorescence polarization dynamics enables simultaneous measurement of the chitinase and lectin activities of lysozyme, which is crucial for its detection in complex biological systems. Hen egg white lysozyme (HEWL), unlike human lysozyme (HL), formed a stable complex with the chitotriose tracer that underwent no further transformations. This fact allows for easy measurement of the carbohydrate-binding activity of the HEWL. The results of the lysozyme activity measurement for hen egg samples obtained through the FPA correlated with the results obtained using the traditional turbidimetry method. The FPA does not have the drawbacks of turbidimetry, which are associated with the need to use bacterial cells that cannot be precisely standardized. Additionally, FPA offers advantages such as rapid analysis, the use of compact equipment, and standardized reagents. Therefore, the new express technique for measuring the lysozyme activity is applicable for evaluating the complex biomaterial, including for the purposes of food product quality control.

## 1. Introduction

Lysozyme, an antimicrobial protein (EC.3.2.1.17), is widely present in various biological tissues, cells, and bodily fluids [1,2]. High concentrations of lysozyme are notably present in egg white [1,2,3]. As a critical component of the immune system in animals, lysozyme facilitates bacterial degradation by phagocytic immune cells, with subsequent bacterial antigen presentation and antibacterial antibody generation [3,4]. Lysozyme belongs to the class of glycoside hydrolases that cleave carbohydrate chains in bacterial cell walls [5,6]. Specifically, lysozyme targets the peptidoglycan (PG) layer of the bacterial cell wall [3,6,7]. PG consists of glycan chains composed of alternating residues of N-acetylglucosamine (NAG) and N-acetylmuramic acid (NAM), cross-linked by peptides attached to the lactyl group of NAM. Lysozyme catalyzes the hydrolysis of β-(1→4)-glycosidic bonds between N-acetylmuramic acid and N-acetylglucosamine, ultimately causing bacterial cell lysis. In addition, lysozyme can destroy glycoside bonds in chitin, although it is not as effective as real chitinases. Because of these properties, lysozyme is widely applied in the food and pharmaceutical industries [3,7,8,9] and medicine [10,11]. Various types of animal lysozymes are described, which have differences in amino acid sequence, structure, physicochemical, and immunologic properties. The main types of lysozymes of animal origin are C-type lysozymes (of the hen type that are found in all mammals), G-type (goose type), and I-type (invertebrate type) [12,13]. The C-type includes hen egg white lysozyme (HEWL) and human lysozyme (HL). Over the past 30 years, they have been commonly used as model systems to study the structure, function, and stability of proteins [14,15].

Lysozyme is effective mainly against Gram-positive bacteria, primarily due to its ability to hydrolyze bonds within the PG layer of the bacterial cell wall. Lysozyme also demonstrates activity against certain Gram-negative bacteria, although these organisms possess an additional outer membrane shielding the PG layer [16,17]. An alternative bacteriolytic mechanism of lysozyme involves its cationic nature, which allows it to disrupt the negatively charged bacterial cell membrane [18,19]. Currently, new aspects of the biological role of lysozyme are under active investigation, including its carbohydrate-binding ability to recognize bacterial antigenic polysaccharides [20]. Lysozyme has also been shown to possess antiviral, antifungal, anticancer, and immunomodulatory properties [1,2,21,22,23]. Monitoring of lysozyme levels is important for the diagnosis of various diseases [2,22].

Evaluation of the bacteriolytic activity of lysozyme requires accurate methods for determining its enzymatic activity. Microbiological count of living bacteria is the straightforward approach to measuring the bacteriolytic activity [24,25]; however, this technique is not accurate, and is time-consuming (10 h and more). Currently, the most widely used method for assessing lysozyme activity in various research and industrial applications is the turbidimetric assay, which measures the rate of absorbance change for bacterial cell suspension in the presence of lysozyme [26,27]. However, full standardization of this method is challenging, as the analytical signal depends on bacterial cell properties, which can vary due to various influencing factors. Fluorescence assay of lysozyme activity is a highly sensitive and reliable method to determine lysozyme activity for some systems of cell culture in which the concentration of lysozyme is relatively low [27,28].

In recent times, for the purposes of determining lysozyme as a protein, various highly sensitive test systems were designed based on DNA-aptamers and printed electrodes modified by carbon nanotubes [27], including a fluorophore of graphene oxide and salicylic phenylacetylene and a DNA-aptamer [28], a FRET biosensor [29], a screen-printed electrode modified by immobilized carbon nanofibers [30] based on surface-enhanced Raman scattering and using the molecular imprinting technique [31,32] or an aptamer [33], a method using HPLC [34], an immunochemical technique using laser nephelometry [35], and an enzyme immunoassay [36,37]. Nonetheless, these methods can only determine the content of lysozyme in total and not its activity. In addition to active lysozyme, the listed methods can detect inactivated lysozyme as well and can potentially provide a false positive result and reveal, for instance, lysozyme-like proteins as fertilization factors [38,39].

The fluorescence polarization assay (FPA) is commonly used to study interactions of biomolecules in a solution [40,41,42]. FPA is based on changes in the rate of rotational diffusion for the fluorescently labeled molecule when its molecular weight increases through interaction with another molecule. Assay efficiency depends on the size of substances taking part in interaction [42]. FPA is also successfully applied to determine enzyme activities [43,44,45]. Thus, the FP assay was introduced to determine lysozyme activity for diagnostic purposes using a fluorescein-labeled natural peptidoglycan of complex structure as the substrate [46,47].

Modern techniques of carbohydrate chemistry allow the synthesis of labeled glycoconjugates [48,49] from oligosaccharides of any structural complexity. These synthetic derivatives are closely related to bacterial [50,51] and fungal [52,53,54] polysaccharide antigens, and their use instead of naturally occurring macromolecules enhances the reproducibility and sensitivity of the immunological and enzymatic testing procedures [55,56].

Based on this technique, we have developed a novel analytical method for determining human lysozyme activity [57]. The method is based on the formation and subsequent disintegration of the enzyme complex with a synthetic chito-oligosaccharide tracer with a rigidly defined structure. Chito-oligosaccharides are β-(1→4)-linked oligomers of N-acetyl-D-glucosamine representing the fragments of peptidoglycan and the polysaccharide chitin. They can be obtained from natural chitin by enzymatic or chemical hydrolysis and subsequent chromatography purification or can be synthesized. Unlike heterogenic fluorescein-labeled natural peptidoglycan derivatives, synthetic fluorescein-labeled chito-oligosaccharides are individual and easy to standardize, ensuring reproducibility of the results with their use.

Despite the fact that the FPA is known for the determination of active lysozyme, it was based on the use of heterogenic fluorescently labeled peptidoglycan samples (see above), which are difficult for standardization. The purpose of this work was to study the applicability of synthetic fluorescein-labeled chito-oligosaccharides for the determination by FPA of active lysozyme in complex biopreparations in the example of chicken egg protein and to compare the obtained data with the results of activity measurement by the usual turbidimetric method based on the lysis rate of whole bacterial cells.

## 2. Materials and Methods

### 2.1. Materials

Synthetic FITC-labeled chitotriose **1**, chitopentaose **2**, and chitoheptaose **3** were obtained from parent spacered oligosaccharides [58], as described earlier [57]. The following commercially available reagents were used: hen egg lysozyme (Sigma, Darmstadt, Germany), lyophilized live bacterial cells Micrococcus luteus, Coomassie G-250 (Sigma-Aldrich, Darmstadt, Germany), Na_2_HPO_4_, NaH_2_PO_4_, NaCl, NaOH, Na_2_CO_3_, citric acid, CuSO_4_ (Component-reagent, Moscow, Russia), C_2_H_5_OH (Bryntsalov, Moscow, Russia), and 70% orthophosphoric acid (Panreac, Barcelona, Spain). Hen eggs were purchased in supermarkets in Moscow, produced by various manufacturers. Egg categories: CB—very large (>75 g); C0—large (from 65 to 74.9 g); C1—medium (55–64.9 g); C2—small (45–54.9 g); 2Y—double-yolk eggs. Physiological solution was used to dilute egg white to 1:10 for the purposes of further analysis.

### 2.2. Active Lysozyme Determination by the Method of Turbidimetry

Lysozyme activity was determined by the lysis rate of the bacterial cells *Micrococcus luteus*, measuring the absorption decrease rate of cell suspension at the wavelength of 650 nm and the temperature of 25 °C [59]. In order to dilute the lysozyme solutions and measure their activity, the 10 mM Na_2_HPO_4_-NaH_2_PO_4_, 150 mM NaCl pH 7.0 buffer that roughly corresponds to the ionic composition of hen egg white was used. The initial absorption of 0.70–0.72 was obtained by adding 35 μL of the stock cell suspension containing 2 mg of the bacterial preparation to 1 mL of the solution. The analytical curve, representing the rate of decrease in absorbance as a function of HEWL concentration, exhibited a linear relationship within the enzyme concentration range of 0 to 5 μg/mL. For lysozyme determination by turbidimetric analysis, all measurements were performed in a 1 mL Shimadzu UV-1801 spectrophotometer cuvette with an optical path length of 10 mm.

### 2.3. Polarization Fluorescence Assay

Working solutions of the fluorescent-labeled oligosaccharide conjugates were prepared in the 10 mM phosphate buffer containing 0.15 M NaCl (pH 7.4). Fluorescence intensity of the solutions exceeded the background signal 10-fold, and amounted to approximately 240,000 units, which corresponds to the 3 nM tracer concentration. Next, 100 μL of the standard hen lysozyme were added to 900 μL of the working solutions to obtain the final concentrations within the range of 10 to 100 μg/mL. The reaction mixture was stirred, and the fluorescence polarization signal was measured every 20 s for 10 min.

The FP dependencies obtained on time for different lysozyme concentrations were approximated using the equation:

mP = mP_0_ + (mP_max_ − mP_0_) × e^−kx^ (1)

where mP is the measured FP signal, mP_0_ is the free tracer FP, mP_max_ is the fluorescence polarization signal for the tracer with lysozyme at the initial moment of time after mixing and k is the observed rate of dissociation of tracer with HEWL at the concentration of HEWL tested. Then, the dependence of k on the lysozyme concentration was plotted.

To determine HEWL using the fluorescence polarization (FP) technique, 100 μL of a 10-fold diluted egg white sample was added to 900 μL of 0.025 M phosphate buffer (pH 7.4) containing 0.15 M NaCl (PBS). Then, FP signals were recorded every 20 s over a 10-min period. The obtained values were fitted according to Equation (1), and lysozyme concentration was calculated based on the calibration curve using the k coefficients. Each test was repeated 3 times for accuracy. FP measurements were conducted with a Sentry-200 instrument (Ellie LLC, Germantown, WI, USA in a tube made from borosilicate glass (10 × 75 mm), λex = 485 and λem = 535 nm. The obtained data were processed using the Sigma Plot 11 software package (Systat Software Inc., Palo Alto, CA, USA).

### 2.4. Total Protein Determination

Quantification of globular non-glycosylated proteins was conducted using the Bradford assay, based on the binding of Coomassie G-250 dye [60]. Total protein and peptide content was measured via the biuret micromethod [61], utilizing a modified Benedict’s reagent [62]. Statistical analysis was performed using Student’s *t*-test, applying a confidence interval of 95%. Each measurement was repeated three times, with results expressed as the mean value. The variability in measurements is indicated on the graphs as standard deviation.

## 3. Results

**Figure 1:**
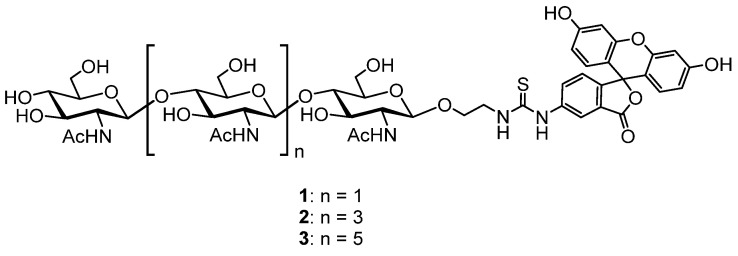
Structure of oligosaccharide tracers **1–3**.

Synthetic fluorescently labeled chito-oligosaccharides **1**–**3** (Figure 1), previously synthesized in studies aimed at quantifying human lysozyme activity [57], were utilized as tracers for measuring active lysozyme in hen egg white using the fluorescence polarization assay (FPA).

**Figure 2:**
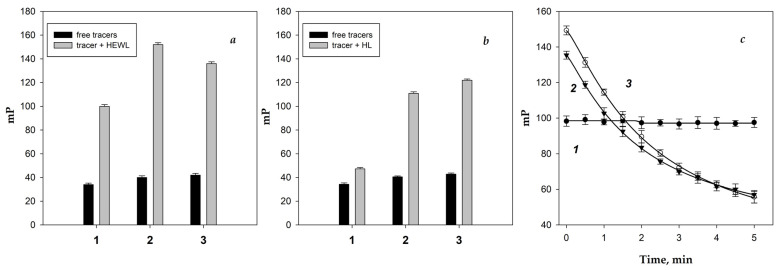
Change in the FP signal of fluorescently labeled conjugates **1**–**3** in presence of hen egg white lysozyme (HEWL) (**a**) and human lysozyme (HL) (**b**). The black bars represent the fluorescence polarization (FP) of working solutions containing tracers **1**–**3** in the absence of proteins, while the gray bars indicate the FP immediately after the addition of the analyte with lysozyme. A significant increase in FP for tracer **1** was observed only after the addition of HEWL, but not HL. The time-dependent dynamics of the FP signal were monitored for tracers **1**–**3** in the presence of HEWL (**c**). The FP signal remained consistently high for tracer **1** but decreased over time for tracers **2** and **3**.

Fluorescence polarization assay tracer solutions **1**–**3** were prepared to achieve fluorescence intensity 10 times higher than the background signal, corresponding to a concentration of 3 nM. The initial FP signal (mP_0_) for the tri-, penta-, and heptasaccharide conjugates (**1**, **2**, and **3**) measured 34.2 ± 1.2, 40.5 ± 0.8, and 42.8 ± 1.0, respectively. Upon addition of 100 μL of hen egg white lysozyme (HEWL) solution at a final concentration of 3 mg/mL, a significant increase in FP signal was observed for all tracers, indicating binding to the enzyme (Figure 2a). In our previous work [57] we showed that fluorescently labeled chito-oligosaccharides **1**–**3** specifically bind exclusively to lysozyme. Notably, in similar assays with human lysozyme (HL), only tracers **2** and **3** showed a marked increase in FP signal, while tracer **1** did not (Figure 2b), suggesting greater stability of the **1**-HEWL complex compared to **1**-HL. The time-dependent dynamics of FP signal changes varied among HEWL complexes with tracers **1**–**3** (Figure 2c). For trisaccharide tracer **1**, FP remained constant over time, indicating that the enzyme did not cleave this tracer. Conversely, FP for penta- and heptasaccharide tracers **2** and **3** decreased rapidly to the initial mP_0_ value, implying substrate cleavage [57].

**Figure 3:**
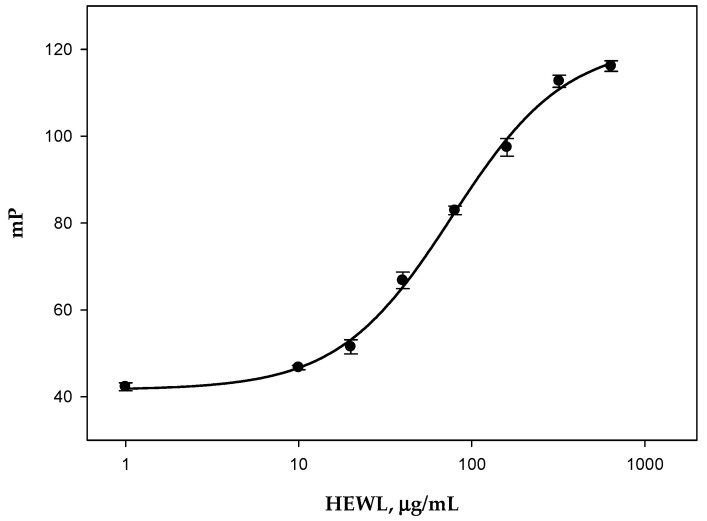
FP signal of tracer **1** in the presence of different amounts of HEWL and approximation calibration curve for the variation in fluorescence polarization (mP) as a function of HEWL concentration. IC_50_ = 76.9 ± 6.5 mg/mL; Hillslope = −1.3 ± 0.1, R^2^ = 0.99.

Trisaccharide **1** has a sufficient size for binding to lysozyme but yet is too short to be split by lysozyme because of its chitinase activity. The unique ability of trisaccharide **1** to bind to HEWL but not to enter into the enzymatic reaction makes it possible to use this tracer to measure the carbohydrate-binding activity of HEWL. When increasing the HEWL concentration from 0 to 640 μg/mL (final concentrations in the cuvette) in the mixture with the tracer **1**, we observed a dose-dependent rise in the FP signal (Figure 3). Based on the experimental results, the FP dependence on the HEWL concentration (calibration curve) was obtained using a semi-logarithmic scale for the HEWL concentration, which was approximated by a four-parameter sigmoid function (2) using Sigma Plot 11 software (Systat Software Inc., San Jose, CA, USA):

(2) $$mP={mP}_{0}+\frac{{mP}_{max}-{mP}_{0}}{{\left(1+\frac{\left[HEWL\right]}{IC50}\right)}^{Hillslope}}$$

where mP is the measured FP signal, mP_0_ is the FP of free tracer, mP_max_ is the fluorescence polarization signal for t tracer **1** with lysozyme at the initial moment of time after mixing, IC_50_ is the HEWL concentration at which a 50% decrease in FP occurs, and Hillslope is a measure of sensitivity (i.e., how steep the response curve is). The limit of detection was calculated by the 3σ method and amounted to 6 μg/mL.

**Figure 4:**
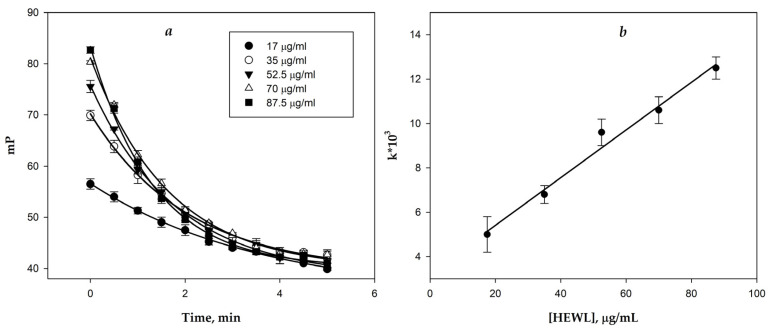
Dynamics of FP signal change in time for tracer **2** in the presence of different amounts of HEWL (**a**); dependence of FP decay rate on HEWL concentration (calibration dependence) and its linear approximation (R^2^ = 0.98) (**b**).

Due to the limited solubility of conjugate **3** in most solvents, which restricts its usability in measurements, and the lack of reactivity between conjugate **1** and HEWL in the enzymatic reaction, the pentasaccharide conjugate **2** was selected for further investigation of lysozyme hydrolytic activity. The kinetics of FP signal variation for tracer **2** were measured for different HEWL concentrations. The kinetic curves (Figure 4a) were fitted according to Equation (1), and the rate constants (k) were calculated for each concentration. Increasing the enzyme concentration produced a dose-dependent increase in the rate of FP signal change. The calibration curve (Figure 4b) demonstrated a linear relationship.

Tracers **1** and **2** were used to define the carbohydrate-binding and hydrolytic activity of lysozyme in hen egg white. The egg white sample was diluted 10-fold in the PBS buffer, followed by adding the tracers **1** or **2** to the working solutions, and measuring the change in FP in time. When the sample interacted with conjugate **1**, an increase in FP was observed right after mixing. Further incubation of the reaction mixture did not result in any change in the FP signal. Using the plotted calibration function (Figure 3), the concentration of lysozyme in eggs was determined by its carbohydrate-binding activity (Table 1, FPA1 column).

Upon the interaction between the hen egg white sample and tracer **2**, an initial increase in the FP signal was observed, followed by a gradual decrease over time. The resulting kinetic profiles were fitted with Equation (1), and the calculated rate constants (k) were applied to determine lysozyme concentrations using the calibration curve on Figure 4b. The results of the hydrolytic activity assay are presented in Table 1, column FPA2. Furthermore, lysozyme levels were measured by traditional turbidimetric analysis based on bacterial lysis. Table 1 also includes data on total peptides and globular protein concentrations, determined by the Bradford protein assay and a biuret micro-method using modified Benedict’s reagent.

The concentrations of active lysozyme, measured by fluorescence polarization analysis using both tracer **1** and tracer **2** are close to each other. The ratio of FPA2 to FPA1 is approximately 1 (Table 1). Comparing the results obtained by FPA and turbidimetry methods, we came to the conclusion that for most egg samples the differences do not exceed the standard error of the test (Table 1). The globular protein to total protein ratio (GP/TG) for all egg samples ranged from 0.16 to 0.33 (16–33% non-glycosylated globular protein to total peptides), indicating that all eggs were of approximately equal quality. The discrepancy was observed for 3 samples (C0-2-6, C0-4-1, CB-5-1) out of 19, where the results of the hydrolytic activity assay (FPA2) and turbidimetry (TM) showed close agreement, while the carbohydrate binding assay (FPA1) gave values overestimating lysozyme activity by 1.5–2 times. For one of these three samples (CB-5-1) we detected a particularly low total peptide and globular protein content. The category of this sample (CB) corresponds to eggs with higher weight, presumably from heavier and older hens. This discrepancy may result from the presence of inactivated lysozyme, which is able to bind the trisaccharide sequence in tracer **1** but lacks the hydrolytic activity.

Across all analyzed samples, the mean lysozyme concentrations determined via FPA 2 and turbidimetry were 3.93 g/L and 3.33 g/L, respectively, yielding an overall average of 3.63 g/L. This corresponds to active lysozyme representing 14% of the total globular, non-glycosylated protein content (26.3 g/L), as measured by Coomassie brilliant blue G-250 dye binding. The average peptide concentration in hen egg white, as measured by the biuret assay, is 124 g/L, indicating that globular proteins constitute only 21% of the peptide content in this biological material. In general, it can be noted that for all egg samples from different producers, such indicators as the content of active lysozyme, globular proteins, and aggregated peptides fall within the reference ranges used for product quality control.

## 4. Discussion

**Figure 5:**
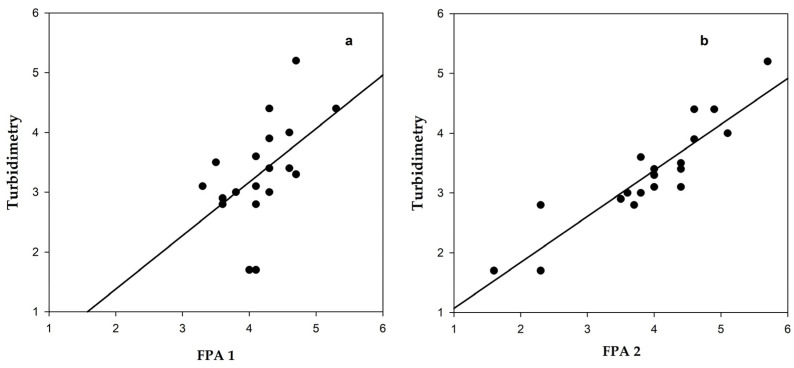
Comparison of FPA methods to determine lysozyme activity using the tracers **1** (**a**) and **2** (**b**), and the traditional turbidimetric method.

The results of active lysozyme determination in hen egg whites using the FPA with pentasaccharide tracer **2** demonstrate a strong correlation with those measured by the traditional turbidimetric method using bacterial cells (Figure 5). Thus, the chitinase activity (ability to cleave chito-oligosaccharides) of lysozyme in complex biological matrices aligns closely with its muramidase activity (ability to cleave peptidoglycan). In addition, FPA analyzing lysozyme using tracer **1**, which measures carbohydrate-binding activity, can provide valuable information about the extent of lysozyme inactivation in complex biological systems, which could be important for assessing food product quality.

Additionally, we demonstrated that lysozymes of different origins, such as HL and HEWL, exhibit distinct interactions with chito-oligosaccharides (Figure 2). This finding enables multiparametric characterization of lysozyme, which can be considered a promising new option for its type-specific identification. Future prospects include the characterization of various lysozyme types (C-type, G-type, and I-type) using chito-oligosaccharides and FPA. This approach has potential applications in assessing the lysozyme composition of biopreparations, such as fungal muramidases, which are widely utilized in agricultural feed production [63].

Table 2 presents the methods for determining lysozyme in hen egg whites, which can be used routinely. Despite the large number of methods proposed for determining lysozyme activity [26,27,28,29,30,31,32,33,34], the classical turbidimetric method is actively used nowadays (Table 2). It is based on the hydrolytic activity of lysozyme leading to the cleavage of glycoside bonds in the peptidoglycan chains of bacterial cell walls. However, a significant disadvantage of the turbidimetric method is its dependence on the quality of used *Micrococcus lysodeikticus* cells, which is almost impossible to standardize. Another common method for assessing lysozyme quality uses chromatographic analysis, but it is not applicable to determine lysozyme enzymatic activity and requires a rather long time for sample preparation and expensive equipment. The use of ELISA to characterize lysozyme activity is also not practical, since it permits the determination of only the total content of lysozyme, and is characterized by a rather wide range of sensitivity and accuracy, as reported by [37,64]. Contrary to the above methods, the approach that is based on determination of chitinase activity of lysozyme by using of PFA and synthetic fluorescently labelled chitooligosaccharide substrates **1** or **2** has several advantages. Particularly, it uses the tracers of distinct structure that permits to run analyses under standard and reproducible conditions. Although FPA has certain limitations, such as the variability of the FP signal with changes in solution viscosity or laboratory temperature, we consider the developed assay a successful alternative to previously reported methods.

## 5. Conclusions

To summarize, two assay formats have been developed for determining the activity of lysozyme: by binding to thrisaccharide tracer **1** (FPA1) and by the rate of hydrolysis of pentasaccharide tracer 2 (FPA2). The advantage of synthetic tracer **1** and **2** is their simple and biosafe synthesis and their high degree of standardization, which ensures reproducibility in assays. The described methods have demonstrated effectiveness in quantifying lysozyme in chicken egg white. Looking forward, analytical approaches based on FPA and chito-oligosaccharide tracers present a promising platform for the profiling and quantification of other lysozyme variants, including those of fungal origin, widely used in the food and agricultural sectors.

## Figures and Tables

**Table 1:** Lysozyme content in the samples of hen egg white, determined by means of FPA and turbidimetry, and the total peptides and the globular proteins.

Egg Sample *	Lysozyme, mg/mL			GP **, mg/mL	TP **, mg/mL	Comparison of Results	
	FPA1 **	FPA2 **	TM **			FPA2/FPA1	GP/TP
2Y-1-1	4.3 ± 0.4	4.6 ± 0.4	3.9 ± 1.0	38 ± 7	114 ± 22	1.07	0.33
2Y-1-2	4.7 ± 0.3	5.7 ± 0.6	5.2 ± 0.5	23 ± 3	133 ± 19	1.21	0.17
2Y-1-3	4.6 ± 0.2	5.1 ± 0.5	4.0 ± 1.0	26 ± 2	131 ± 18	1.11	0.20
C0-2-1	3.6 ± 0.3	3.5 ± 0.3	2.9 ± 0.8	32 ± 3	132 ± 19	0.97	0.24
C0-2-2	5.3 ± 0.5	4.9 ± 0.5	4.4 ± 1.0	32 ± 8	148 ± 24	0.92	0.22
C0-2-3	4.1 ± 0.3	3.8 ± 0.4	3.6 ± 0.8	23 ± 7	117 ± 18	0.93	0.20
C0-2-4	4.3 ± 0.2	4.6 ± 0.4	4.4 ± 0.8	27 ± 2	165 ± 21	1.07	0.16
C0-2-5	3.8 ± 0.4	3.6 ± 0.3	3.0 ± 0.5	21 ± 2	122 ± 15	0.95	0.17
C0-2-6 ***	4.1 ± 0.5	2.3 ± 0.1	1.7 ± 0.6	29 ± 7	118 ± 15	0.56	0.24
C0-3-1	3.3± 0.3	4.0 ± 0.3	3.1 ± 0.4	29 ± 4	116 ± 22	1.21	0.25
C0-3-2	3.5 ± 0.4	4.4 ± 0.4	3.5 ± 0.9	26 ± 3	114 ± 17	1.26	0.23
C0-3-3	4.3 ± 0.5	4.4 ± 0.5	3.4 ± 0.8	25 ± 7	97 ± 13	1.02	0.26
C0-4-1 ***	4.1 ± 0.2	2.3 ± 0.2	2.8 ± 0.6	21 ± 4	131 ± 20	0.56	0.16
CB-5-1 ***	4.0 ± 0.4	1.6 ± 0.2	1.7 ± 0.5	18 ± 3	72 ± 15	0.40	0.25
C2-6-1	4.1 ± 0.2	4.4 ± 0.4	3.1 ± 0.6	23 ± 3	128 ± 17	1.07	0.18
C2-6-2	4.3 ± 0.4	3.8 ± 0.4	3.0 ± 0.5	26 ± 3	130 ± 23	0.88	0.20
C2-6-3	4.7 ± 0.3	4.0 ± 0.3	3.3 ± 0.5	29 ± 5	133 ± 15	0.85	0.22
C2-6-4	4.6 ± 0.2	4.0 ± 0.5	3.4 ± 0.5	26 ± 4	136 ± 21	0.87	0.19
C1-7-1	3.6 ± 0.3	3.7 ± 0.3	2.8 ± 1.0	26 ± 4	127 ± 16	1.03	0.20

* Egg samples are coded as XX-Y-Z where XX is egg category, Y is producer number, Z is egg number. Egg categories: CB—very large (>75 g); C0—large (from 65 to 74.9 g); C1—medium (55–64.9 g); C2—small (45–54.9 g); 2Y—double-yolk eggs. *** 3 samples with diverging measurement results (see the explanations in text). ** Abbreviations: FPA1—developed assay that quantifies the increase in the fluorescence polarization (FP) signal of tracer **1** upon the addition of the analyte. This assay is designed to assess the carbohydrate-binding activity of lysozyme in the sample. FPA2—developed assay that quantitatively measures the rate of decrease in the fluorescence polarization (FP) signal following the initial increase induced by the addition of tracer **2**. This assay is designed to evaluate the hydrolytic (chitinase) activity of lysozyme in the sample. TM—turbidimetry. GP—globular proteins. TP—total peptides.

**Table 2:** Characteristics of common methods for lysozyme determination and here-described approach.

Method	Recognition Element	LOD *	Detection Range	Analysis Time	Ref.
Turbidimetry	*Micrococcus lysodeikticus*	1.94 µg/mL	NS **	10 min	[34]
HPLC	Acetonitrile gradient in 0.1% trifluoroacetic acid.	3.84 µg/mL	NS	40 min	[34]
ELISA	MAb ***	0.264 µg/mL	0.38–4.8 µg/mL	2–3 h	[37]
FPA	Fluorescein labelled chitooligosacharides	6 µg/mL	20–90 µg/mL	5 min	This work

* LOD—limit of detection, ** NS—not specified, *** MAb—monoclonal antibody.

## Data Availability

Data are available from the corresponding author.
